# Antidepressant Intake and Recovery of Dysphagia After Acute Ischemic Stroke

**DOI:** 10.1161/STROKEAHA.125.054073

**Published:** 2026-02-13

**Authors:** Anel Karisik, Kurt Moelgg, Lucie Buergi, Lukas Scherer, Luisa Delazer, Benjamin Dejakum, Silvia Felicetti, Christian Boehme, Thomas Toell, Raimund Pechlaner, Simon Sollereder, Sonja Rossi, Michael Thomas Eller, Gudrun Schoenherr, Wilfried Lang, Johann Willeit, Peter Willeit, Stefan Kiechl, Michael Knoflach, Lukas Mayer-Suess, on behalf of the STROKE-CARD Study Group, Markus Anliker

**Affiliations:** Department of Neurology (A.K., K.M., L.B., L.S., L.D., B.D., S.F., C.B., T.T., R.P., M.T.E., G.S., J.W., S.K., M.K., L.M.-S.), Medical University of Innsbruck, Austria.; ICONE-Innsbruck Cognitive Neuroscience, Department for Hearing, Speech and Voice Disorders (S.R.), Medical University of Innsbruck, Austria.; Institute of Clinical Epidemiology, Public Health, Health Economics, Medical Statistics and Informatics (P.W.), Medical University of Innsbruck, Austria.; VASCage-Centre on Clinical Stroke Research, Innsbruck, Austria (A.K., K.M., L.B., L.S., S.S., W.L., S.K., M.K.).; Medical Faculty, Sigmund Freud Private University, Vienna, Austria (W.L.).; Department of Public Health and Primary Care, University of Cambridge, United Kingdom (P.W.).

**Keywords:** antidepressive agents, deglutition disorders, ischemic stroke, pneumonia, aspiration, quality of life

## Abstract

**BACKGROUND:**

Poststroke dysphagia is associated with poor functional recovery and psychological consequences, including depression and fatigue, which may impede successful rehabilitation. Here, we investigate whether antidepressants may improve dysphagia recovery after acute ischemic stroke.

**METHODS:**

In this prospective cohort study, patients with acute ischemic stroke (aged ≥18 years; consecutively enrolled in the STROKE-CARD trial (Post-Stroke Disease Management) 2014 to 2019 and registry 2020 to 2023 in Innsbruck, Austria) were examined for poststroke dysphagia (by standardized clinical and instrumental examinations) and antidepressant intake at hospital admission, discharge, and inpatient 3-month follow-up. The outcome was full oral diet resumption 3 months poststroke. Associations were analyzed using multivariable logistic regression and are presented as adjusted odds ratios, adjusting for age, sex, stroke severity, dysphagia severity, depression severity, stroke localization, thrombolysis, cognitive impairment, functional disability before stroke and at hospital discharge, and study type.

**RESULTS:**

Poststroke dysphagia affected 380 (18.6%) of the total cohort of 2046 patients at hospital admission (mean age, 72.7±14.1 years; 37.8% women) and persisted in 290 (14.7%) and 95 (4.6%) patients until hospital discharge and 3-month follow-up, respectively. Among the 290 patients with persistent dysphagia at discharge included in the outcome analysis, antidepressant intake increased from 4.8% before stroke to 27.6% at hospital discharge and 49.0% at 3 months (*P*<0.001). A total of 195 (67.2%) of 290 patients with persistent dysphagia regained full oral diet by 3 months, with significantly higher recovery rates in those who were discharged on antidepressants (78.8% versus 62.9%; *P*=0.010). Antidepressant intake at hospital discharge was independently associated with improved dysphagia recovery at 3 months (adjusted odds ratio, 2.98 [95% CI, 1.51–5.87]; *P*=0.002).

**CONCLUSIONS:**

Antidepressant intake was associated with better dysphagia recovery after acute ischemic stroke. As these findings are observational, randomized trials are required to clarify whether antidepressants can support dysphagia rehabilitation.

Poststroke dysphagia is a frequent and serious complication of acute ischemic stroke, contributing to malnutrition, aspiration, pneumonia, and increased mortality.^1–3^ Beyond its medical consequences, poststroke dysphagia significantly impairs quality of life, often leading to social isolation and reduced participation in daily activities.^4,5^

Emerging evidence links dysphagia to a heightened risk of poststroke depression and fatigue, both affecting up to 33% and 50% of stroke survivors in the first year.^6–9^ These complications can severely hinder rehabilitation, consequently worsening functional outcomes and diminishing quality of life.^10–12^ While treatment options for poststroke fatigue remain limited, antidepressant intake, primarily selective serotonin reuptake inhibitors (SSRIs), has been widely studied in the poststroke depression setting.^13,14^ While some studies suggest a potential role in enhancing functional recovery after stroke, recent meta-analyses found no consistent benefit for overall functional outcome, motor deficits, or aphasia in stroke populations.^14,15^ However, whether antidepressant intake specifically supports recovery of poststroke dysphagia remains unclear.

## Aims and Hypothesis

1. This study aims to evaluate the potential impact of antidepressant intake on the recovery of dysphagia in patients with acute ischemic stroke.
2. Our specific objective was to assess the association between antidepressant intake and the course of dysphagia recovery in a consecutive cohort of patients with ischemic stroke.

## Methods

### Data Availability

Anonymized patient data are available to qualified researchers upon reasonable request (lukas.mayer@i-med.ac.at). Access will be granted following review and approval of a research proposal with a corresponding statistical analysis plan and the execution of a formal data-sharing agreement.

### Patient Cohort

We analyzed data from 2 consecutive stroke cohorts: the STROKE-CARD trial (Post-Stroke Disease Management; 2014–2019; Unique identifier: NCT02156778) and the STROKE-CARD registry (2020–2023; Unique identifier: NCT04582825), both conducted at the University Hospital of Innsbruck, Austria. The hospital serves as the comprehensive stroke center for ≈900 000 inhabitants and the primary hospital for 2 districts covering 300 000 inhabitants.

Both studies applied the STROKE-CARD program, a poststroke disease management initiative inviting all adult (aged ≥18 years) consecutive ischemic stroke and high-risk patients with transient ischemic attack to structured and standardized outpatient visits at the study center at 3 and 12 months. The effect of the STROKE-CARD program was initially evaluated in a randomized-controlled trial (STROKE-CARD trial) and later implemented as a standardized care framework and prospective registry (STROKE-CARD registry).^16,17^

The sole difference between both studies was that the STROKE-CARD trial excluded patients with a life expectancy <1 year, drug or alcohol addiction, or severe disability with poor rehabilitation prognosis (modified Rankin Scale [mRS] score of 5 at discharge), leading to the exclusion of 2% (n=60), 1% (n=35), and 9% (n=243) of otherwise eligible patients, respectively.^17,18^ The STROKE-CARD registry had no exclusion criteria.

**Figure 1.**
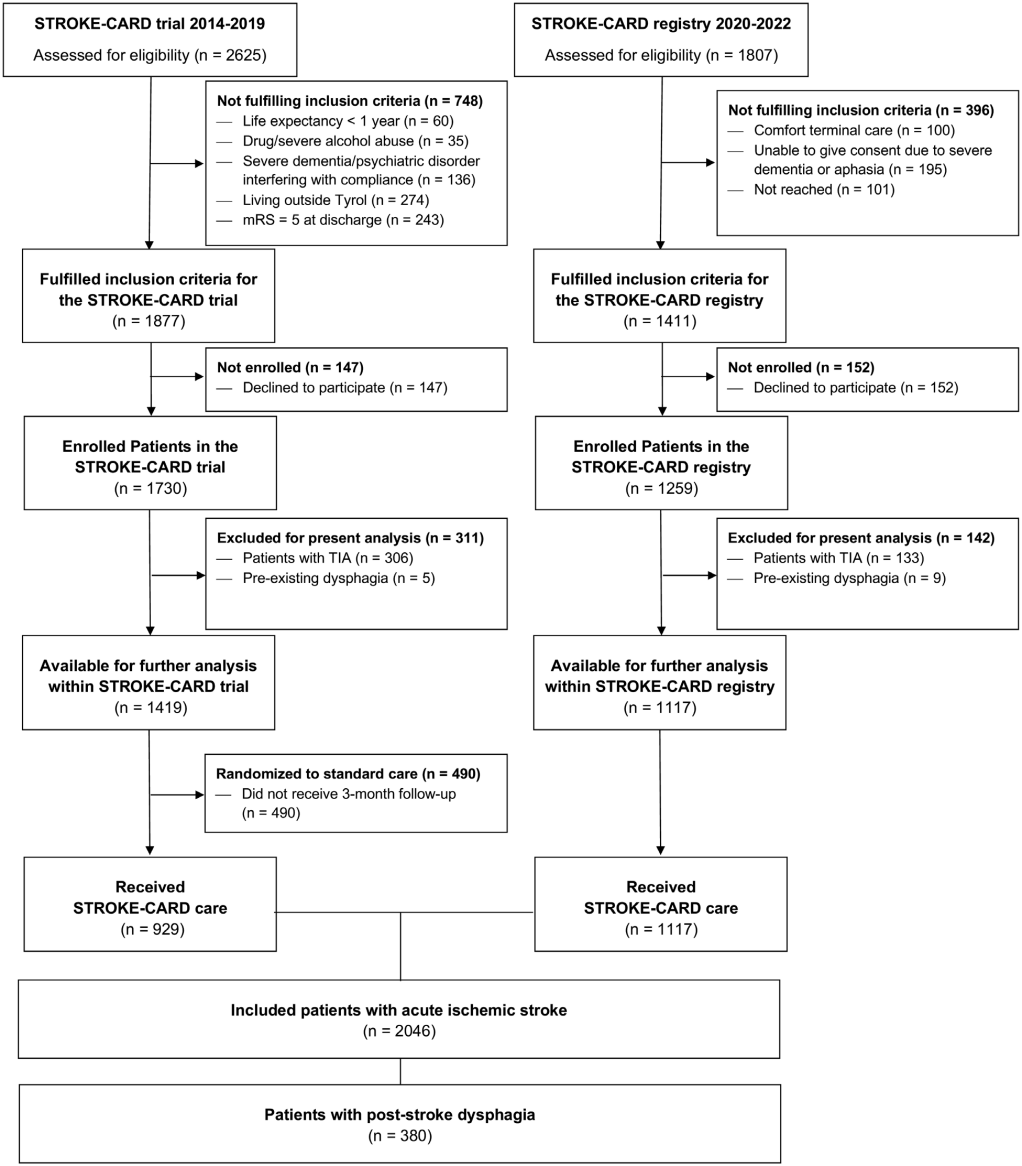
**Study flowchart.** mRS indicates modified Rankin Scale; STROKE-CARD, Post-Stroke Disease Management; and TIA, transient ischemic attack.

For this analysis, we included all patients with consecutive ischemic stroke within the STROKE-CARD program (2014–2019 for the trial and 2020–2023 for the registry; Figure 1). Both studies were approved by the local ethics committee, and all participants provided written informed consent. Detailed study protocols are available elsewhere.^16,17^ This study was conducted and reported in accordance with the STROBE guidelines (Strengthening the Reporting of Observational Studies in Epidemiology; Supplemental Material).

### Data Collection

Dysphagia screening and diagnosis followed a predefined 3-step protocol within the entire cohort. Initial screening was systematically performed within 24 hours of admission, if clinically possible, using the Gugging Swallowing Screen. If dysphagia was suspected, patients underwent a comprehensive clinical swallowing assessment by speech and language therapists. In the case of inconclusive findings or suggested severe dysphagia, fiberoptic endoscopic evaluation of swallowing was conducted. Swallowing impairment was categorized according to the validated Functional Oral Intake Scale (FOIS) into mild (requiring minor dietary modifications, avoiding mixed consistencies; FOIS, 5–6), moderate (significant dietary adjustments, prohibiting thin liquids; FOIS, 3–4), and severe (necessitating oral feeding only under supervision or nothing by mouth; FOIS, 1–2).^19^ Persistent dysphagia was defined as the inability to return to a full oral diet at both hospital discharge and the 3-month follow-up (FOIS < 7). All swallowing evaluations and FOIS classifications were performed by certified speech and language therapists as part of routine clinical care; assessors were not formally blinded to medication lists.

Antidepressant intake was documented at 3 time points: prestroke, at hospital discharge, and at the 3-month follow-up. Drugs were categorized as SSRIs (mainly citalopram and sertraline), selective serotonin-norepinephrine reuptake inhibitors (including duloxetine and venlafaxine), others (noradrenergic and specific serotonergic antidepressants or tricyclics, including mirtazapine and amitriptyline), or a combination therapy.

Concerning patient characteristics, stroke location was classified into anterior (infarcts in the middle cerebral artery, anterior cerebral artery, or border-zone regions), posterior (infarcts affecting the posterior cerebral artery, cerebellum, or brainstem), or both territories combined. Stroke severity was assessed using the National Institutes of Health Stroke Scale score at admission. Acute revascularization measures undertaken (eg, intravenous thrombolysis) were recorded. Functional status was measured using the mRS before the stroke, at hospital discharge, and at 3-month follow-up. Cognitive impairment was determined based on documented clinical diagnoses in medical records. Depressive symptoms after stroke were assessed by the Beck Depression Inventory, categorized into minimal (scores of 0–9, including patients without completed questionnaires [<10%] if neither they nor their relatives reported symptoms), mild (scores of 10–18), moderate (scores of 19–29), and severe (scores of 30–63) symptoms.^20^

Within this analysis, patients with poststroke dysphagia at discharge were considered as our study cohort with the primary outcome of our analysis being set as full oral diet resumption 3 months poststroke.

### Statistical Approach

Baseline characteristics were summarized using means with SDs or medians with interquartile ranges for continuous variables and frequencies for categorical variables. Group differences between patients with and without antidepressant intake were assessed using the χ^2^ tests for categorical data and the Mann-Whitney U tests for continuous variables. Changes in antidepressant use across 3 time points (prestroke, hospital discharge, and 3-month follow-up) among patients with persistent dysphagia were analyzed using the Cochran Q test, followed by pairwise McNemar tests with Bonferroni correction for post hoc comparisons. To evaluate the association between antidepressant intake and dysphagia recovery, multivariable logistic regression models were applied, estimating adjusted odds ratios while controlling for potential confounders. Confounding variables were selected for inclusion in the multivariable model based on prior literature and retained if they were significant (*P*<0.05) in univariate analysis. Conclusively, 3 models applying the following confounders were used: (1) model 1: age, sex, prestroke mRS score, cognitive impairment, National Institutes of Health Stroke Scale score at baseline, and thrombolysis; (2) model 2: all factors of model 1 and adjustment for anterior circulation stroke, severe dysphagia at baseline, type of study (ie, STROKE-CARD trial or registry), moderate to severe depression poststroke (Beck Depression Inventory >18), and inability to walk (mRS score ≥4) at hospital discharge; and (3) model 3: all factors of model 2 including persistent inability to walk at 3-month follow-up (mRS score ≥4). Bonferroni correction was applied to adjust for multiple comparisons. Sensitivity analyses were conducted to assess the effect of antidepressant intake in a restricted cohort of patients without prestroke antidepressant exposure or who had died before the 3-month follow-up. To account for changes in antidepressant use over time, a per-protocol analysis was conducted, only including patients who remained not treated with antidepressants throughout the 3-month follow-up in the no antidepressant group (results are provided in Table S1). Statistical significance was set at *P*<0.05.

## Results

### Baseline Characteristics and Dysphagia Prevalence

Of the 2046 patients included (mean age, 72.7±14.1 years; 37.8% women; Figure 1), poststroke dysphagia was present in 380 (18.6%) patients at admission. Among them, 188 (49.5%) had mild (FOIS, 5–6), 151 (39.7%) had moderate (FOIS, 3–4), and 41 (10.8%) had severe dysphagia (FOIS, 1–2). Persistent dysphagia was evident in 290 (14.7%) at hospital discharge and 95 (4.6%) at 3-month follow-up.

The following analyses focus on our study cohort, those with dysphagia at hospital discharge (n=290; Table 1). The table additionally presents the patient characteristics in group comparison between those with and without antidepressant intake at hospital discharge.

**Table 1.**
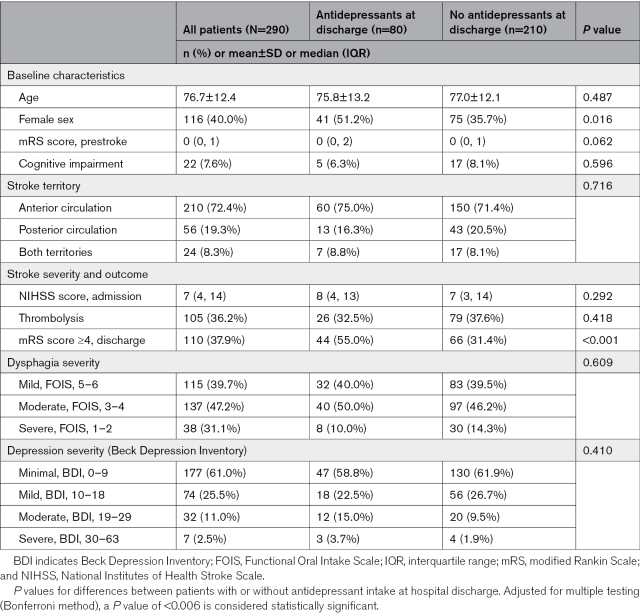
Baseline Characteristics of Patients With Persistent Poststroke Dysphagia Stratified by Antidepressant Intake at Hospital Discharge

In short, the mean age within our cohort of interest was 76.7±12.4 years, and 40.0% were women. Cognitive impairment at baseline was found in 7.6% of patients. Most strokes occurred in the anterior circulation (72.4%), and 36.2% of patients received thrombolytic therapy. Median National Institutes of Health Stroke Scale score at admission was 7 (4–14), and 37.9% of patients had severe disability (mRS score ≥4) at hospital discharge. In subgroup comparison, after adjusting for multiple testing, only the frequency of mRS score ≥4 at discharge differed between those with (80, 27.6%) and those without (210, 72.4%) antidepressant intake at discharge. Patients with antidepressant intake were also more commonly women; this, however, did not reach statistical significance. There was no difference in dysphagia severity and depression scores between the groups.

### Antidepressant Intake and Dysphagia Recovery

In total, 142 of 290 (49.0%) patients with dysphagia at discharge took antidepressants at 3-month follow-up. Antidepressant intake increased substantially over time: from 4.8% prestroke to 27.6% at discharge and 49.0% at the 3-month follow-up (*P*<0.001; detailed characteristics shown in Table 2).

**Table 2.**
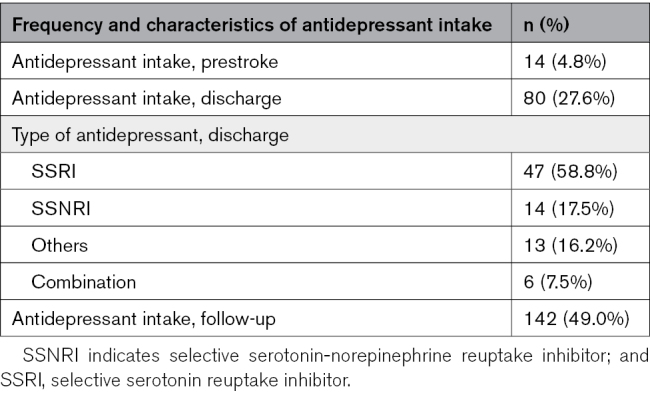
Need for Antidepressants Before and After Acute Ischemic Stroke

SSRIs were the most prescribed class (58.8%, n=47), followed by selective serotonin-norepinephrine reuptake inhibitors at 17.5% (n=14), and other antidepressants at 16.2% (n=13); 7.5% (n=6) received combination therapy.

Of the patients started on antidepressants before discharge (n=80), nearly all (98.7%, n=79) remained on the same treatment until the 3-month follow-up. Prestroke antidepressant use was similar between patients discharged on antidepressants and those without antidepressant intake (6.3% versus 4.3%; *P*=0.485).

**Figure 2.**
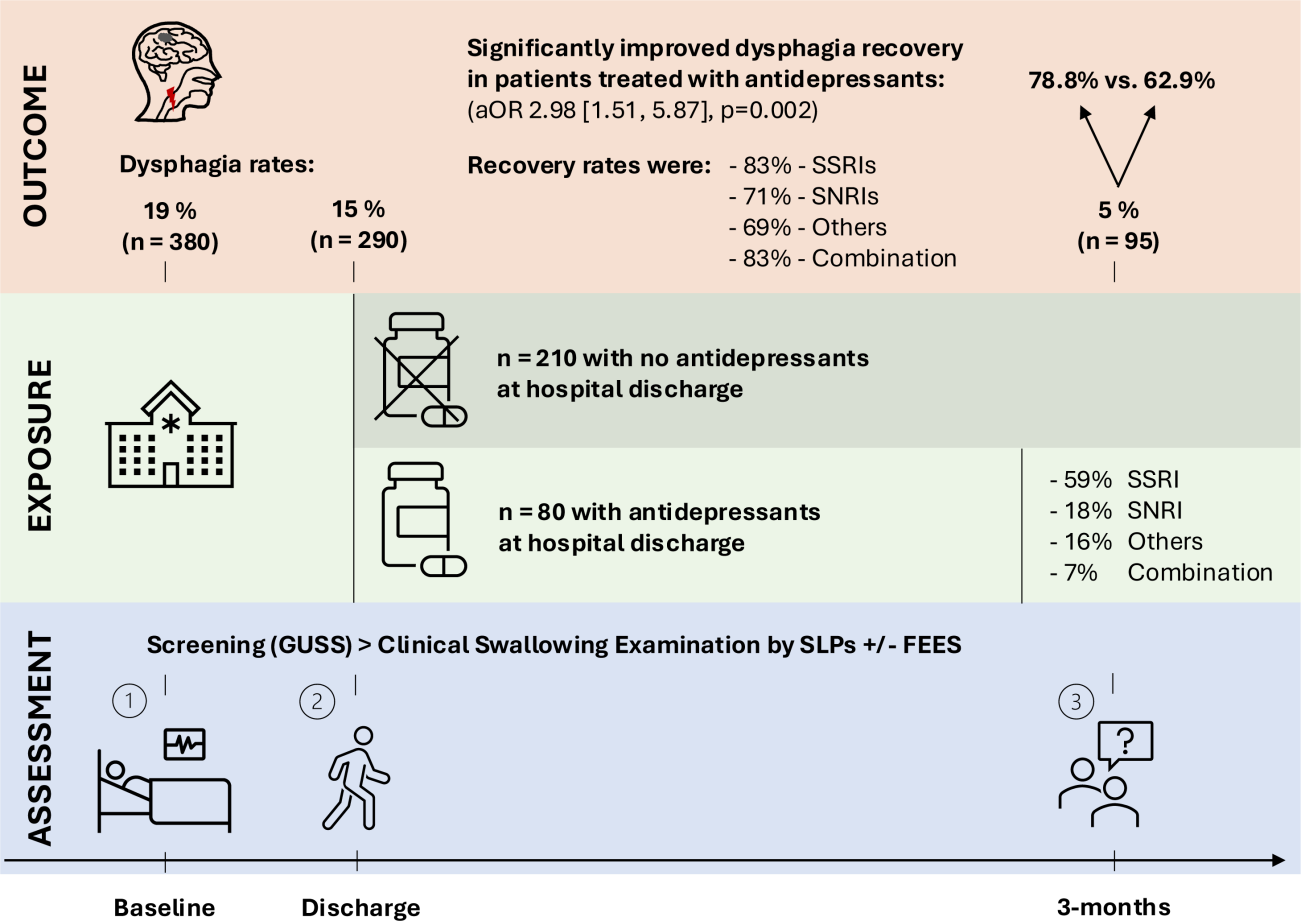
**Dysphagia recovery in relation to antidepressant exposure.** aOR indicates adjusted odds ratio; FEES, fiberoptic endoscopic evaluation of swallowing; GUSS, Gugging Swallowing Screen; SLP, speech and language therapist; SNRI, selective serotonin-norepinephrine reuptake inhibitor; and SSRI, selective serotonin reuptake inhibitor.

At 3-month follow-up, 195 (67.2%) individuals with poststroke dysphagia at discharge achieved full oral diet resumption by 3 mo. Thirteen patients died before the 3-month follow-up; all had regained swallowing function before death. Recovery rates from dysphagia were significantly higher among those who had antidepressant treatment at discharge compared with those who did not (78.8% vs 62.9%; *P*=0.010; Figure 2).

Among antidepressant subclasses, recovery rates were 83% for SSRIs, 71% for SNRIs, 69% for other antidepressants, and 83% for combination therapy. All subclasses showed higher recovery rates than patients without antidepressant treatment (62.9%), with SSRIs and combination therapy demonstrating the highest rates of recovery.

Antidepressant intake at discharge was independently associated with improved dysphagia recovery at 3 months (adjusted odds ratio, 2.98 [95% CI, 1.51–5.87]; *P*=0.002), after adjusting for age, sex, stroke severity, dysphagia severity, stroke localization, depression severity, thrombolysis, cognitive impairment, functional disability at discharge, and type of study. Findings were independent of overall functional recovery (mRS) at 3-month follow-up (adjusted odds ratio, 2.74 [95% CI, 1.41–5.32]; *P*=0.003). Exclusion of patients who died before the 3-month follow-up or with prestroke antidepressant intake yielded similar results (shown in Table 3). Furthermore, the association was further strengthened when patients who initiated antidepressants after hospital discharge were excluded (shown in Table S1). Adding dysarthria or angiotensin-converting enzyme inhibitor intake to the multivariable analysis did not alter the association between antidepressant intake and dysphagia recovery (data not shown).

**Table 3.**
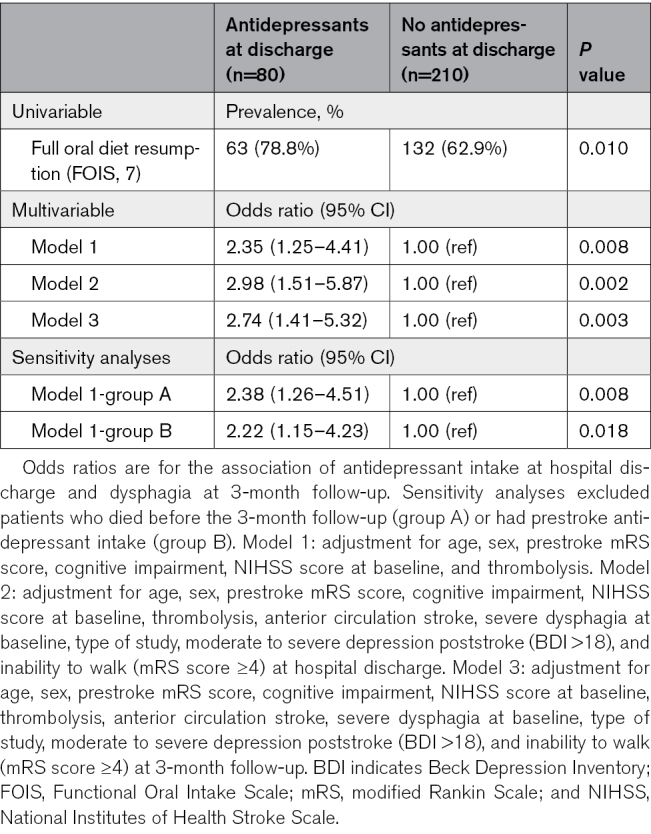
Association Between Antidepressant Intake and Dysphagia Recovery

## Discussion

This is the first study that demonstrates an independent association between antidepressant intake and improved dysphagia recovery following acute ischemic stroke. Patients with antidepressant intake at hospital discharge exhibited significantly higher rates of full oral diet resumption at 3 months poststroke, which was independent of overall poststroke functional recovery, but associated with antidepressant intake at hospital discharge.

While previous studies have investigated the role of antidepressants in poststroke motor, aphasia, and overall functional recovery, their specific impact on dysphagia recovery has been largely overlooked.^14,15^ To date, only one retrospective study has been conducted and showed a potential benefit of fluoxetine on dysphagia recovery after stroke.^21^ Our findings support the possibility that antidepressants may exert selective effects on specific functional domains, such as swallowing, rather than producing broad improvements across all stroke-related impairments. This is particularly clinically relevant, as several meta-analyses have demonstrated that SSRIs are associated with an increased risk of adverse events, including falls, fractures, and seizures.^14,15,22^ Therefore, the decision to initiate antidepressant therapy in stroke patients must be made cautiously, with careful consideration of individual risk profiles. Identifying patients at high risk for poststroke depression that may be causally linked to clinical symptoms that benefit from antidepressant treatment, that is, poststroke dysphagia as shown by our analysis, will be essential for maximizing therapeutic gains while minimizing harm.

The observed association between antidepressants and dysphagia recovery may be explained by multiple mechanisms. Antidepressants, particularly SSRIs, have been shown to potentially enhance motor recovery by modulating serotonergic pathways involved in cortical reorganization.^23^ Because swallowing recovery has been linked to the activation and reorganization of compensatory neuronal areas in the unaffected hemisphere, SSRIs may, though speculative, offer a beneficial effect.^24,25^ While evidence for SNRIs and other antidepressants is limited, agents such as venlafaxine have demonstrated increased motor excitability and improved performance in motor tasks in healthy adults, suggesting a potential role in supporting recovery.^26^ Beyond direct neuroplastic effects, antidepressants may counteract the negative impact of poststroke depression and fatigue, which might impair motivation and participation in rehabilitation.^27^ By improving mood and reducing fatigue, patients may be more engaged in swallowing therapy, ultimately facilitating functional recovery.

This study has several notable strengths. The large underlying cohort of patients with consecutive ischemic stroke and the broad spectrum of stroke severity and age groups enhance the generalizability of our findings to a wide stroke population eligible for poststroke care. By leveraging prospectively collected data, we ensured a comprehensive assessment of dysphagia through standardized clinical and instrumental swallowing examinations. In addition, the inclusion of detailed clinical variables allowed for adjustment of potential confounders, strengthening the validity of our results. A supplementary analysis further supported the main findings, showing that the association between antidepressant use and dysphagia recovery was more pronounced when excluding patients who initiated treatment only after hospital discharge.

Certain limitations must be acknowledged. As an observational study, causality cannot be established, and residual confounding remains despite statistical adjustments. Antidepressant intake was not randomized, and detailed information on dose, titration, or adherence was not systematically available. This and the limited sample size among individual antidepressant treatment groups preclude conclusions about specific treatment effects or dose-response relationships. A general risk of confounding by indication exists in nonrandomized treatment studies. In our case, antidepressants were prescribed by physicians for depressive or psychosocial reasons rather than swallowing impairment, and speech and language therapists had no role in this decision-making process. We adjusted for multiple indicators of stroke severity, functional status, cognition, and depression to mitigate this risk. Stratified analyses by dysphagia severity were limited by small subgroup sizes, particularly among patients with severe dysphagia, precluding reliable subgroup-specific estimates. Last, swallowing assessments were not formally blinded to antidepressant intake. These factors highlight the nonrandomized design and support interpreting our findings as hypothesis-generating.

Prior research in this setting has shown that intensified poststroke care improves dysphagia recovery, suggesting that the observed association with antidepressants likely applies within or in combination with structured and intensified rehabilitation efforts.^28^ Furthermore, our study population was recruited in a country with universal health care access, and >99% of patients were of European descent. Findings may not necessarily apply to ethnically more diverse populations or populations with limited health care access or significant heterogeneity in risk factor prevalence and stroke incidence. However, these limitations harbor an opportunity as future randomized trials assessing the effect of antidepressant intake in patients with poststroke dysphagia are needed to confirm our exploratory findings. Such trials may combat a to-date unresolved issue of poststroke dysphagia care and might determine the optimal therapeutic strategy in the future. These data may serve as a quantitative basis for the design of such trials in the future.

## Conclusions

Within our observational study of patients with consecutive ischemic stroke, antidepressant intake was associated with improved dysphagia recovery upon 3-month follow-up. Randomized-controlled trials are warranted to validate this exploratory association and to determine whether antidepressants may have a role as an adjunct to dysphagia rehabilitation.

## Article Information

### Acknowledgments

The authors thank the Speech and Language Therapy Team at the Department of Neurology, Medical University of Innsbruck, Austria. Gudrun Schoenherr, MSc, died.

### Disclosures

Dr P. Willeit reports compensation from Novartis for consultant services. The other authors report no conflicts.

### Supplemental Material

Table S1

STROBE Checklist

## Supplementary Material



## Appendix

The STROKE-CARD Study Group: Markus Anliker, Gregor Broessner, Julia Ferrari, Martin Furtner, Julian Granna, Andrea Griesmacher, Ton Hanel, Viktoria Hasibeder, Katharina Kaltseis, Gerhard Klingenschmid, Theresa Köhler, Stefan Krebs, Florian Krismer, Clemens Lang, Christoph Mueller, Wolfgang Nachbauer, Anna Neuner, Anja Perfler, Thomas Porpaczy, Gerhard Rumpold, Christoph Schmidauer, Theresa Schneider, Eva Wagner, Lisa Seekircher, Uwe Siebert, Christine Span, Martin Sojer, Lydia Thiemann, Lena Tschiderer, Marlies Wichtl, and Karin Willeit.
