# Supplementary material for: Antidepressant Intake and Recovery of Dysphagia After Acute Ischemic Stroke
Source: Stroke. 2026 Feb 13;57(4):1000–7. doi: 10.1161/STROKEAHA.125.054073 (PMC13003936; doi:10.1161/STROKEAHA.125.054073)
Supplement: Supplementary file 1 [file str-57-1000-s001.pdf]

Antidepressant intake and recovery of dysphagia after acute ischemic stroke

SUPPLEMENTAL MATERIAL

Table S1: Association between antidepressant intake and dysphagia recovery (excluding patients with antidepressant initiation after hospital discharge)

	Antidepressants at discharge (n=80)	No antidepressants at discharge (n=147)	
**Univariable**	**Prevalence, %**		**p value**
Full oral diet resumption (FOIS 7)	66 (79.5%)	84 (58.3%)	0.001
**Multivariable**	**Odds ratio (95% CI)**		**p value**
Model 1	2.91 [1.50, 5.64]	1.00 (ref.)	0.002
Model 2	4.37 [2.05, 9.33]	1.00 (ref.)	<0.001
Model 3	3.77 [1.83, 7.79]	1.00 (ref.)	<0.001
**Sensitivity Analyses**	**Odds ratio (95% CI)**		**p value**
Model 1 – Group A	2.86 [1.45, 5.61]	1.00 (ref.)	0.002
Model 1 – Group B	2.62 [1.31, 5.25]	1.00 (ref.)	0.007

Odds ratios are for the association of antidepressant intake at hospital discharge and dysphagia at 3-month follow-up. Sensitivity analyses excluded patients who died before 3-month follow-up (Group A) or had pre-stroke antidepressant intake (Group B).

OR - Odds Ratio, CI - Confidence interval, mRS - modified Rankin Scale, NIHSS - National Institutes of Health Stroke Scale, FOIS – Functional Oral Intake Scale.

Model 1: Adjustment for age, sex, pre-stroke mRS, cognitive impairment, NIHSS at baseline and thrombolysis.
Model 2: Adjustment for age, sex, pre-stroke mRS, cognitive impairment, NIHSS at baseline, thrombolysis, anterior circulation stroke, severe dysphagia at baseline, type of study, moderate to severe depression post-stroke (BDI > 18), and inability to walk (mRS ≥ 4) at hospital discharge.
Model 3: Adjustment for age, sex, pre-stroke mRS, cognitive impairment, NIHSS at baseline, thrombolysis, anterior circulation stroke, severe dysphagia at baseline, type of study, moderate to severe depression post-stroke (BDI > 18), and inability to walk (mRS ≥ 4) at 3-month follow-up.
